# On the contrasting morphological response to far-red at high and low photon fluxes

**DOI:** 10.3389/fpls.2023.1185622

**Published:** 2023-06-02

**Authors:** Paul Kusuma, Bruce Bugbee

**Affiliations:** ^1^Department of Plant Sciences, Horticulture and Product Physiology, Wageningen University and Research, Wageningen, Netherlands; ^2^Crop Physiology Laboratory, Department of Plants Soils and Climate, Utah State University, Logan, UT, United States

**Keywords:** far-red, phytochrome, shade avoidance, shade tolerance, photosynthetic photon flux density, leaf expansion

## Abstract

Plants compete for sunlight and have evolved to perceive shade through both relative increases in the flux of far-red photons (FR; 700 to 750 nm) and decreases in the flux of all photons (intensity). These two signals interact to control stem elongation and leaf expansion. Although the interacting effects on stem elongation are well quantified, responses for leaf expansion are poorly characterized. Here we report a significant interaction between far-red fraction and total photon flux. Extended photosynthetic photon flux density (ePPFD; 400 to 750 nm) was maintained at three levels (50/100, 200 and 500 µmol m^-2^ s^-1^), each with a range of 2 to 33% FR. Increasing FR increased leaf expansion in three cultivars of lettuce at the highest ePPFD but decreased expansion at the lowest ePPFD. This interaction was attributed to differences in biomass partitioning between leaves and stems. Increased FR favored stem elongation and biomass partitioning to stems at low ePPFD and favored leaf expansion at high ePPFD. In cucumber, leaf expansion was increased with increasing percent FR under all ePPFD levels showing minimal interaction. The interactions (and lack thereof) have important implications for horticulture and warrant further study for plant ecology.

## 1 Introduction

Limited resources limit plant growth (El-Sharkawy, 2011). As sessile organisms, plants must modulate their growth and development to compete for access to critical resources – one of which is sunlight. This has led to the evolution of a high degree of plant plasticity in response to shaded environments.

Before plants experience a decrease in the flux of photons within photosynthetically active radiation (PAR; 400 to 700 nm) in competitive environments, the flux of far-red photons (FR; 700 to 750 nm) will increase due to reflection from neighboring vegetation primarily in the horizontal direction (Ballaré et al., 1987; Ballaré et al., 1991; Casal, 2012). This increase in FR (decrease in the R:FR ratio) is caused by preferential absorption of photons in the PAR region for photosynthesis and scattering of FR photons by leaf tissue. As plants grow, they begin to overlap, which decreases the overall flux of photons in both the PAR and FR regions, although the percentage of FR [100×FR/(PAR + FR)] remains enriched compared to sunlight. Many plant species adjust their morphology in response to both FR and PAR to maximize survival in a process called shade avoidance (Ballaré et al., 1991; Schmitt, 1997; Kurepin et al., 2007a; Pierik and de Wit, 2014). Shade avoidance responses are often defined as increased stem and petiole elongation, increased biomass allocation to stems, increased specific leaf area (SLA; leaf area divided by leaf mass), and increased apical dominance (Casal, 2012; Gommers et al., 2013; Pierik and de Wit, 2014).

Biomass accumulation is highly correlated with leaf area, especially before canopy closure when plants are young and a high fraction of photons fall between the plants (Monteith, 1977; Gifford et al., 1984; Zhen and Bugbee, 2020b). Furthermore, leaves are the primary photon-collecting organs of a plant with higher photosynthetic capacity than other organs (Xu et al., 1997). Increasing leaf area increases the area over which photons can be intercepted, and thus, leaf area/expansion in response to shade is a vital parameter for the competitiveness, survival, and fitness of a plant. Specific leaf area, which is often reported to increase in shade, only indicates a change in leaf area given the biomass partitioning to the leaves. Thus, total plant leaf area is dependent on both the SLA and the biomass partitioning, the latter of which tends to favor stems over leaves in shaded environments (Morgan and Smith, 1979; Ballaré et al., 1987; Ballaré et al., 1991; Poorter et al., 2012).

The literature has been inconsistent regarding the effect of increasing FR on leaf expansion. We summarize these contrasting responses across 41 studies spanning 46 years in **Table S1**. Within these 41 studies, 14 show that increasing percent far-red or end-of-day far-red can decrease leaf area (Holmes and Smith, 1975; Holmes and Smith, 1977; Frankland and Letendre, 1978; Child et al., 1981; Kwesiga and Grace, 1986; Robson et al., 1993; Devlin et al., 1999; Franklin et al., 2003; Carabelli et al., 2007; Kurepin et al., 2007a; Kurepin et al., 2007b; Qaderi et al., 2015; Park and Runkle, 2019; Kong and Nemali, 2021), 28 studies show increases (Child et al., 1981; Kwesiga and Grace, 1986; Casal et al., 1987; López-Juez et al., 1990; Pausch et al., 1991; Heraut-Bron et al., 1999; Franklin et al., 2003; Kurepin et al., 2006; Kurepin et al., 2007a; Boccalandro et al., 2009; Kurepin et al., 2012a; Kurepin et al., 2012b; Patel et al., 2013; Lee et al., 2016; Shibuya et al., 2016; Bae et al., 2017; Park and Runkle, 2017; Park and Runkle, 2018; Kalaitzoglou et al., 2019; Park and Runkle, 2019; Shibuya et al., 2019; Zou et al., 2019; Zhen and Bugbee, 2020b; Jin et al., 2021; Kong and Nemali, 2021; Kusuma and Bugbee, 2021; Legendre and van Iersel, 2021; Zou et al., 2021), and 14 studies show no effect (Child et al., 1981; Pausch et al., 1991; Barreiro et al., 1992; Miyashita et al., 1995; Heraut-Bron et al., 1999; Kurepin et al., 2006; Kurepin et al., 2010; Kurepin et al., 2012b; Patel et al., 2013; Lee et al., 2015; Park and Runkle, 2017; Park and Runkle, 2018; Park and Runkle, 2019; Zhang et al., 2020). These contrasting responses are well noted in the literature (see Casal and Smith, 1989; Casal, 2012; Demotes-Mainard et al., 2016), and have been attributed to species/cultivar differences (Casal et al., 1987; Boccalandro et al., 2009; Kurepin et al., 2012b), as well as interactions with vapor pressure deficit (Shibuya et al., 2019), temperature (Franklin et al., 2003; Patel et al., 2013), blue photon flux (Park and Runkle, 2019), and PAR intensity (Kurepin et al., 2007a; Park and Runkle, 2018; Zou et al., 2019).

One confounding factor with many of these studies is that FR photons are usually added to a constant flux of traditionally defined PAR. Recent evidence has demonstrated that FR photons are photosynthetic when combined with shorter wavelengths (Zhen and Bugbee, 2020a; Zhen and Bugbee, 2020b) through excitation of PSI (Zhen and van Iersel, 2017). Because many studies add FR on top of traditionally defined PAR, the flux of total photosynthetic photons is increased in these studies. This potentially leads to an increase in leaf area through an increase in photosynthesis and biomass accumulation rather than through a morphological adjustment to increase light capture. It is thus important to properly separate the two effects. The conclusion that FR is photosynthetic has resulted in the development of the term extended photosynthetically active radiation (ePAR), which considers the photons between 400 to 750 nm photosynthetic (Zhen et al., 2021). This metric has been increasingly adopted over the past few years (Kusuma and Bugbee, 2021; Shelford and Both, 2021; Veazie et al., 2023; Warner et al., 2023). Maintaining extended photosynthetic photon flux density (ePPFD; the photon flux of ePAR; 400 to 750 nm), while adjusting levels of FR, is one way to separate the photosynthetic and morphological responses.

Plant morphological responses to increases in FR are modulated by phytochrome photoreceptors (Holmes and Smith, 1975; Franklin et al., 2003; Casal, 2012), while responses to decreases in ePPFD are modulated by phytochromes (Millenaar et al., 2009; Trupkin et al., 2014), cryptochromes (Keller et al., 2011; Pedmale et al., 2016), and possibly photosynthetic signals (Millenaar et al., 2009). Cryptochromes and phytochromes interact with many of the same transcription factors to modulate gene expression (de Wit et al., 2016), but cryptochrome effects (low blue photon fluxes) have been shown to regulate genes related to cell wall elasticity, while FR was associated with auxin genes (Pedmale et al., 2016). Phytochrome responses depend on the ePPFD because thermal reversion (the light independent conversion of Pfr back to Pr) significantly affects phytochrome dynamics at lower photon fluxes (Sellaro et al., 2019). Additionally, hormonal signals in response to FR have different patterns of expression at low and high ePPFD (Hersch et al., 2014).

Altogether, the signaling pathways in response to changes in photon quality and quantity are complex, as photoreceptor-controlled responses have appeared both additive/independent (Neff and Chory, 1998; Keller et al., 2011; Pedmale et al., 2016) but also synergistic (Casal and Mazzella, 1998; Sellaro et al., 2009; de Wit et al., 2016). It has been hypothesized that one of the reasons FR decreases leaf expansion in some species is because of competition for resources between stems and leaves (Casal et al., 1987). If FR and PAR interact to control stem elongation, and if increased stem elongation induces more biomass partitioning to the stems (de Wit et al., 2018), then FR and PAR ought to be expected to interact to predict leaf expansion, potentially showing opposite responses to FR at high and low ePPFD. The response may depend on the basic architecture of the specific species, especially regarding rosette versus upright plant architecture.

We investigated the interactions of FR and ePPFD on plant morphology, with specific interest in leaf expansion, in two diverse species, lettuce and cucumber (rosette and upright plant architecture, respectively). We hypothesize that increasing the percentage of FR will have contrasting effects at different levels of ePPFD. We show that increasing FR in lettuce increases leaf area and dry mass accumulation at high intensities but decreases leaf area and dry mass accumulation at lower intensities, while in cucumber leaf expansion increased with increasing FR at all intensities.

## 2 Materials and methods

### 2.1 Plant material and cultural conditions

Green butterhead lettuce (*Lactuca sativa* ‘Rex’) and cucumber (*Cucumis sativa* ‘Straight Eight’) seeds were direct seeded then thinned for uniformity after emergence leaving four plants per root module. Two supplemental studies with lettuce cultivars ‘Waldmann’s Dark Green’ (replicated once) and ‘Green Salad Bowl’ (replicated three times) were conducted following the same methods as ‘Rex’ lettuce to determine the consistency of the responses across cultivars. Root modules measured 20 × 18 × 13 cm (4680 cm^3^) and contained a 1:1 ratio of peat and vermiculite by volume with 0.75 g per L wetting agent (AquaGro G), 1 g per L hydrated lime, and 1 g per L of uniformly mixed slow-release fertilizer (Nutricote total; 18-6-8, N-P-K, type 70). Planted root modules were randomly placed into the 12 treatment chambers. Each chamber had dimensions of 20 × 23 × 30 cm (L × W × H, 13800 cm^3^) with gloss white walls. Fans provided an air velocity of 0.5 m s^-1^ at the top of the canopy. Root modules were watered to 10% excess as needed with dilute fertilizer solution at a rate of 100 mg N per L (21-5-20; Peters Excel; EC = 1 dS m^-1^) and allowed to passively drain. Type-E Thermocouples connected to a data logger (CR1000, Campbell Scientific, Logan UT) continuously monitored ambient air temperature. Day/night temperature averaged 23.4 ± 1.2/20.9 ± 0.8 ˚C, with less than a 1˚C variation among chambers. CO_2_ concentration was continuously monitored and was identical for all treatments and varied over time between 450 to 500 ppm.

### 2.2 Treatments

**Figure 1:**
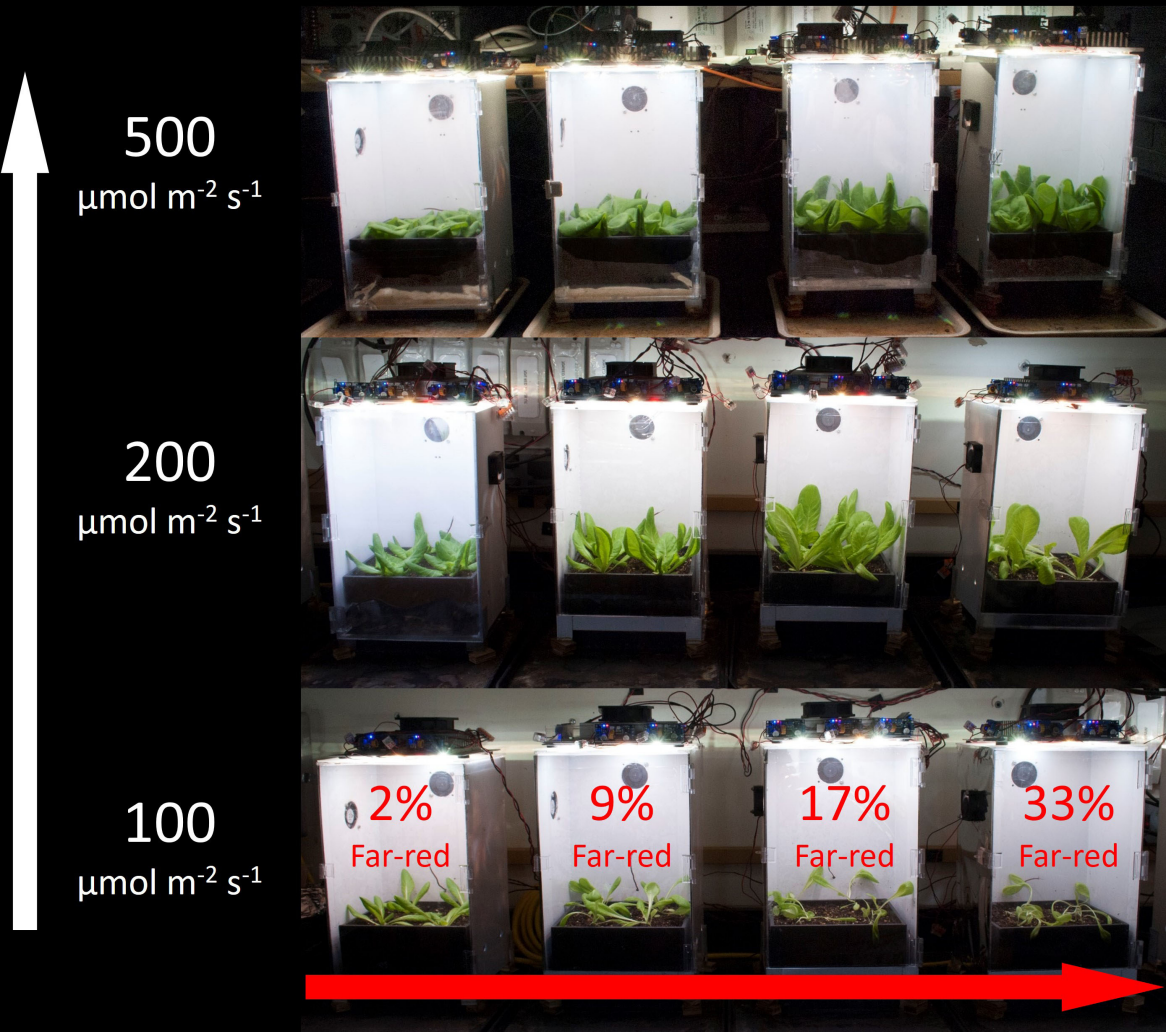
Experimental design of the study. Treatments included four percentages of far-red at three levels of extended photosynthetic photon flux density (ePPFD). The lowest ePPFD in the cucumber study was 50, instead of 100, µmol m^-2^ s^-1^.

**Figure 2:**
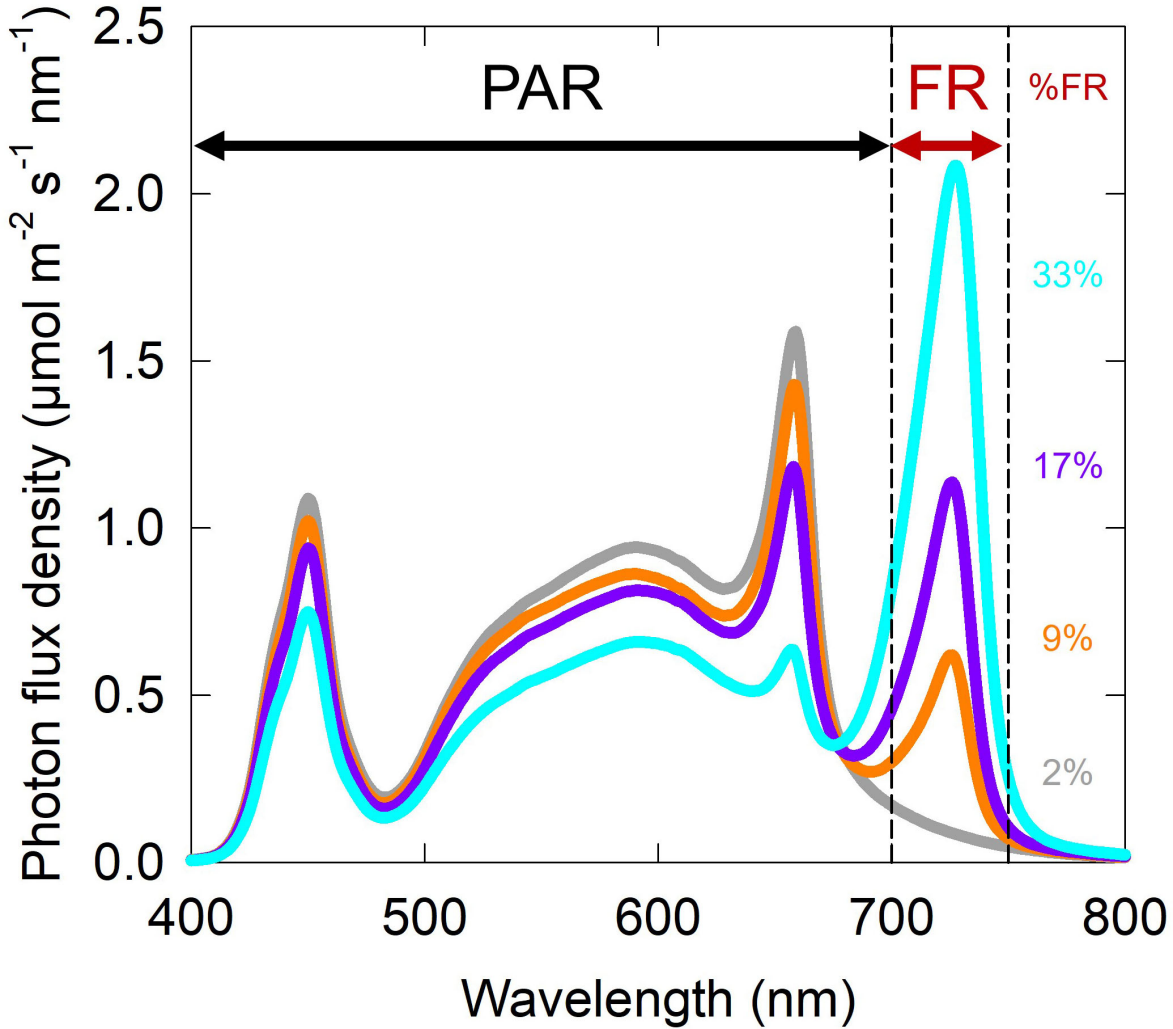
Representative spectral photon distributions for the four fractions of far-red (FR) from the ePPFD = 200 µmol m^-2^ s^-1^ intensity treatments. Spectral photon distributions from the other two intensities had the same shape with different scales on the y-axis. Photon fluxes of blue, green and red were maintained at 20, 40 and 40%, respectively, as a percent of photosynthetically active radiation (PAR; 400 to 700 nm) for each treatment of FR. Percent FR (%FR) treatments included 2% (grey), 9% (orange), 17% (purple), and 33% (cyan).

The system included 12 chambers with four percentages of FR (**Figures 1**, **2**) at three levels of ePPFD (4×3 = 12 treatments). Light was provided over a 16 h photoperiod. In the cucumber study the three levels of ePPFD were 50, 200 and 500 µmol m^-2^ s^-1^ (Daily light integral, DLI: 2.88, 11.52 and 28.8 mol m^-2^ d^-1^). In the lettuce studies, the lowest ePPFD treatment (50 µmol m^-2^ s^-1^) was increased to 100 µmol m^-2^ s^-1^ (DLI: 5.76 mol m^-2^ d^-1^) because 50 µmol m^-2^ s^-1^ was below the light compensation point.

Treatments were developed using white (2700 K and 6500 K), red (peak about 658 nm) and far-red (peak 726 nm) LEDs (Lumileds LLC, San Jose, CA) to output 20% blue, 40% green, and 40% red photons as a proportion of PAR. Treatments reduced traditionally defined PAR while increasing FR photon flux density. Percent FR, calculated by dividing the FR photon flux density by the ePPFD and multiplying by 100, was 2, 9, 17 and 33% (**Figures 1**, **2**). Spectral photon distributions of the treatments were measured before each replicate study with a spectroradiometer (model PS-300; Apogee Instruments, Logan UT). ePPFD was measured with an ePAR sensor (MQ-610, Apogee Instruments, Logan UT) at the top of the plant canopy throughout the study, and chambers were adjusted to maintain ePPFD at ± 5% of the setpoint. Root modules were rotated every three days to increase the uniformity of treatments within the chamber.

### 2.3 Plant measurements

Plants were harvested at canopy closure. This occurred 17 or 18 days after emergence in lettuce and 9, 10, or 11 days after emergence in cucumber. At harvest, stem length, leaf area, and fresh mass were measured. Leaf area was measured using a leaf area meter (LI-3000; LI-COR, Lincoln NE). Petiole length was measured in the cucumber study and leaf length and leaf width were measured in the lettuce study. Leaves and stems (and cotyledons in cucumber) were separated, and dry mass (DM) of each organ was measured after the tissue was dried at 80 ˚C for 48 hours. Percent stem mass was calculated by dividing stem dry mass by total shoot dry mass, multiplied by 100; roots were not included. Specific leaf area (SLA, m^2^_LA_ kg DM_leaf_^-1^) was calculated by dividing the leaf area by the leaf dry mass.

### 2.4 Statistical analysis

The lettuce study was replicated three times and the cucumber study was replicated five times. Every replicate in time contained four plants in each treatment. All data was analyzed using R statistical software (R Foundation for Statistical Computing; Vienna, Austria). ePPFD was treated as a categorical variable with three levels for all analysis. Percent FR was treated as either a continuous variable or a categorical variable depending on the general response of the parameter. Responses that were linear with increasing percent FR were analyzed with linear mixed-effects regression analysis (percent FR treated as a continuous variable) and non-linear responses were analyzed with two-way ANOVA analysis (percent FR treated as a categorical variable). Replicates in time were treated as random factors in all statistical analyses. Significant effects were determined at the p < 0.05 level. Detailed statistical analysis for specific parameters are provided below.

## 3 Results

**Figure 3:**
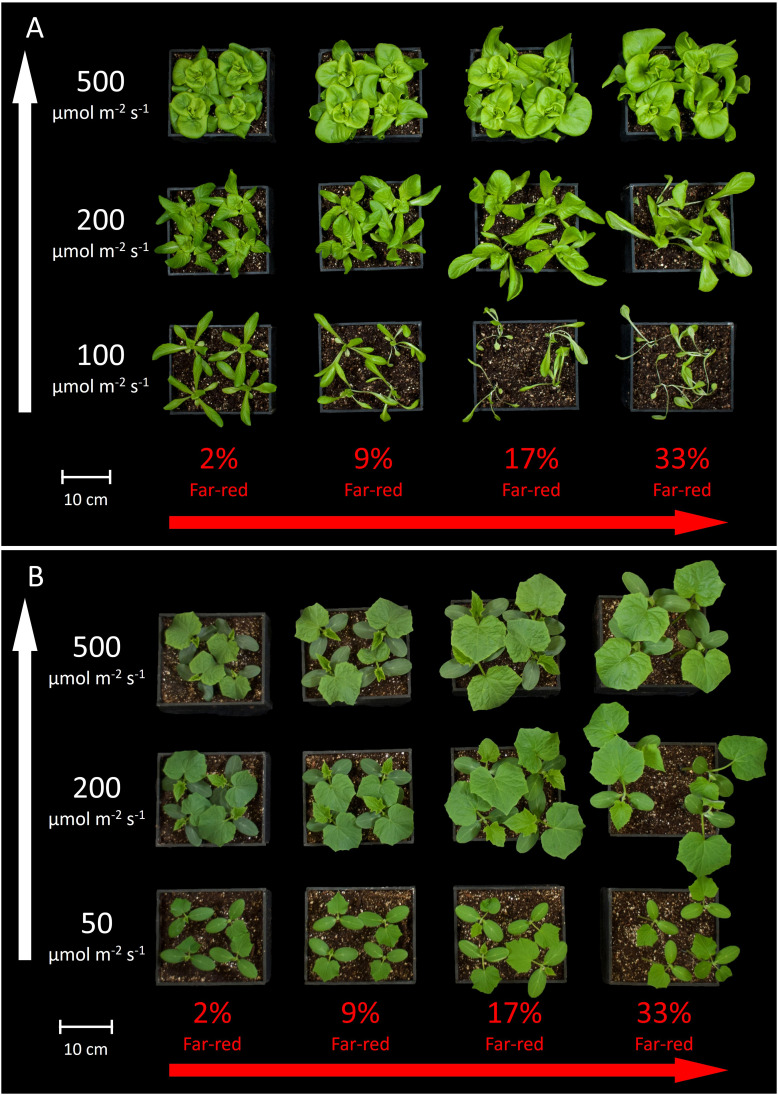
Overhead view of all the treatments in one replicate for **(A)** lettuce and **(B)** cucumber. The white arrow on the left indicates increasing extended photosynthetic photon flux density (ePPFD) and the red arrow on the bottom indicates increasing percent far-red.

**Figure 4:**
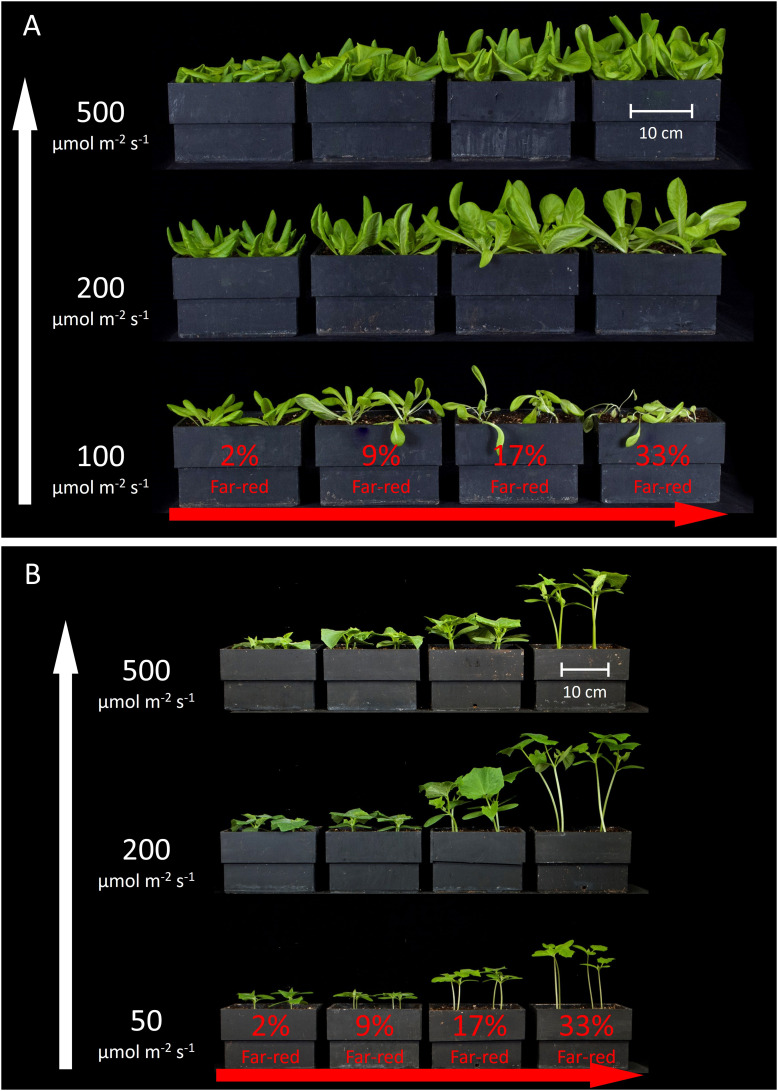
Side view of all the treatments in one replicate for **(A)** lettuce and **(B)** cucumber. The white arrow on the left indicates increasing extended photosynthetic photon flux density (ePPFD) and the red arrow on the bottom indicates increasing percent far-red.

**Figure 3** shows the overhead view of all the plants from each treatment in one replicate study, and **Figure 4** shows the side view of the plants from each treatment in one replicate study. **Figure S1** shows the overhead view of both the ‘Waldmann’s Dark Green’ and ‘Green Salad Bowl’ supplementary lettuce studies. These photos show approximate plant diameters and heights in each treatment.

### 3.1 Biomass accumulation

**Figure 5:**
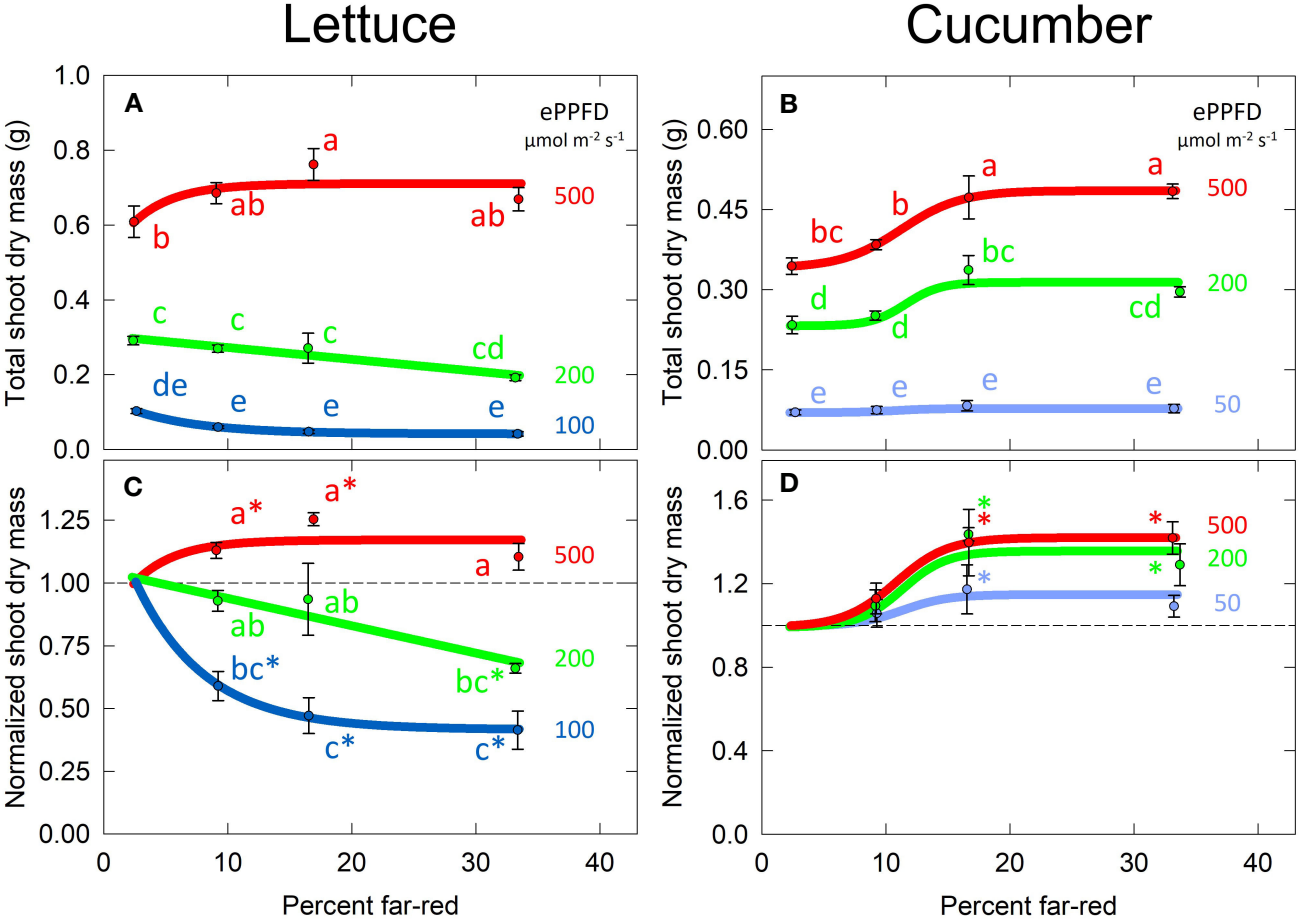
Effects of percent far-red (FR) and extended photosynthetic photon flux density (ePPFD) on shoot dry mass in lettuce **(A, C)** and cucumber **(B, D)**. **(A, B)** represent the original values of dry mass and **(C, D)** are the normalized response, where data from each replicate in time was normalized to its respective 2% FR (no added FR) control treatment for each ePPFD. In **(C, D)**, * indicates that the treatment is statistically different from 1 (using a student’s t-test), which represents the response of the 2% FR control. Trend lines are included to guide the eye, and not meant to be used as a statistical representation. Error bars represent standard error for n = 3 replicates in lettuce and n = 5 replicates in cucumber. Letters are not included in **(D)** because the treatments were determined to be insignificantly different from each other, although several treatments were significantly different from their respective controls.

The effect of percent FR on total shoot dry mass was non-linear in both lettuce and cucumber (**Figures 5A, B****)**, thus two-way ANOVA analysis and Tukey’s *post hoc* test were used (with percent FR as a categorical variable) to separate the significant differences between treatments. The smaller values and variance of total dry mass in the low ePPFD treatment resulted in reduced statistical power to determine significant difference between percent FR treatments. Therefore, the data of each replicate in time was normalized to its respective 2% FR (no added FR) control, and this normalized response was analyzed with 1) two-way ANOVA analysis/Tukey’s *post hoc* test to determine significant differences between treatments and 2) a student’s t-test to determine if the normalized response was statistically different from one, which represented the response of no added FR (**Figures 5C, D****)**.

Higher ePPFD produced more biomass in both species. In lettuce, percent FR and ePPFD interacted to predict biomass accumulation, where increasing percent FR increased dry mass at high ePPFD, but decreased dry mass at low ePPFD. This decrease occurred in both the 100 and 200 µmol m^-2^ s^-1^ ePPFD treatments. There was also an interaction between percent FR and ePPFD in cucumber, where the highest percent FR at the lowest ePPFD had no effect compared to higher ePPFD (**Figure 5D**), but in general the interaction was far less pronounced when compared to lettuce. In general, the response of cucumber shoot dry mass showed no effect of increasing percent FR from 2 to 9%, an increase between 9 and 17% FR, and little response between 17 and 33% FR.

### 3.2 Total leaf area

**Figure 6:**
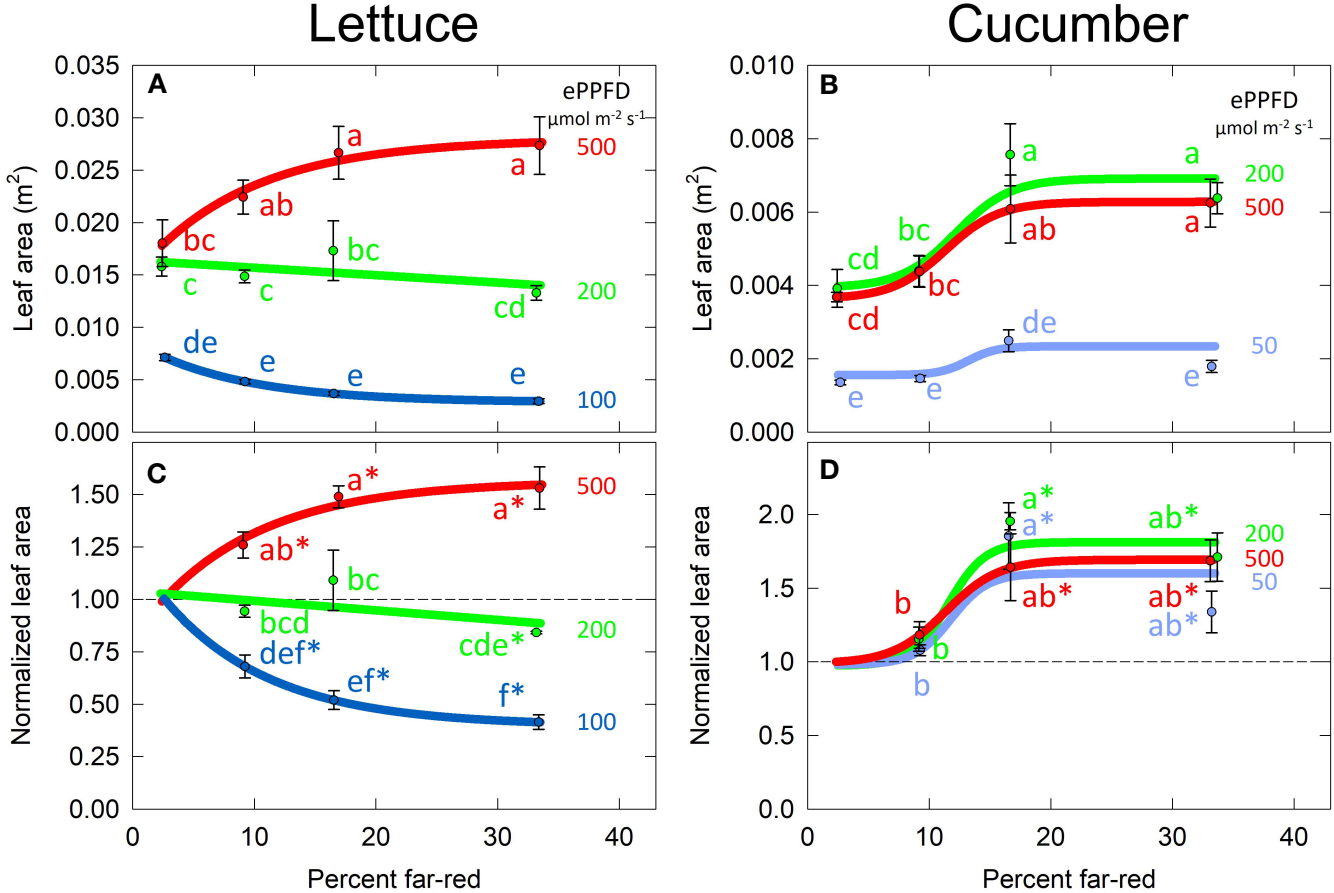
Effect of percent far-red (FR) at different levels of extended photosynthetic photon flux density (ePPFD) on specific leaf area (SLA) in lettuce **(A, C)** and cucumber **(B, D)**. **(A, B)** represent the original values of SLA, while **(C, D)** are the normalized response, where data has been normalized to the average response in the 2% FR control treatment for each ePPFD. Increasing the percent FR and decreasing the ePPFD increased SLA in both species. Error bars represent standard error for n = 3 replicates in lettuce and n = 5 replicates in cucumber.

The effect of percent FR and ePPFD on total leaf area was similar to the effects on shoot dry mass (**Figure 6**). This indicates that the effects of percent FR on biomass accumulation in **Figure 5** were likely driven by the ability of the plant to capture photons (leaf area is highly correlated with growth before canopy closure). Due to the similar non-linear response of leaf area to percent FR and ePPFD, this response was analyzed with the same methods as total dry mass.

Because leaf area/leaf expansion significantly contributes to the rate of biomass accumulation, it is valuable to better understand the interaction between the FR and ePPFD in controlling this response in lettuce. Leaf lengths increased with percent FR at all three levels of ePPFD, while the response of leaf width was similar to the response of leaf area (**Figures S2A, B**). This resulted in longer, narrower leaves rather than rounder leaves at higher percent FR (**Figure S2C**). Although an increase in leaf length is generally expected under shaded conditions, the change in leaf shape does not fully explain the interaction between percent FR and ePPFD in predicting total leaf area in lettuce.

### 3.3 Specific leaf area

Specific leaf area (SLA) is a measure of leaf area given the biomass partitioning to the leaves, and it is widely reported to increase with shade. Compared to the responses of dry mass and total leaf area, the response of SLA to percent FR appeared linear. Thus, this parameter was analyzed with linear mixed-effects regression with percent FR as a continuous variable and ePPFD as a categorical variable. Increasing the percent FR and decreasing the ePPFD increased SLA in both species (**Figures 6A, B****)**.

Because ePPFD had a large effect on SLA (the lowest ePPFD induced a 2.5 to 3 times higher SLA than the highest ePPFD in both species), the best way to determine if there is an interaction between percent FR and ePPFD was to normalize the response. This is because the non-normalized response could indicate an interaction even if the effect of percent FR induced the same fractional change at all three levels of ePPFD. **Figures 6C, D** normalizes the SLA response to the average 2% FR response at each ePPFD. Linear mixed-effects regression analysis was then performed on this normalized data, which found no interaction between ePPFD and percent FR for either species, although the interaction approached significance in cucumber (p = 0.11; **Figure 6D**).

**Figure 7:**
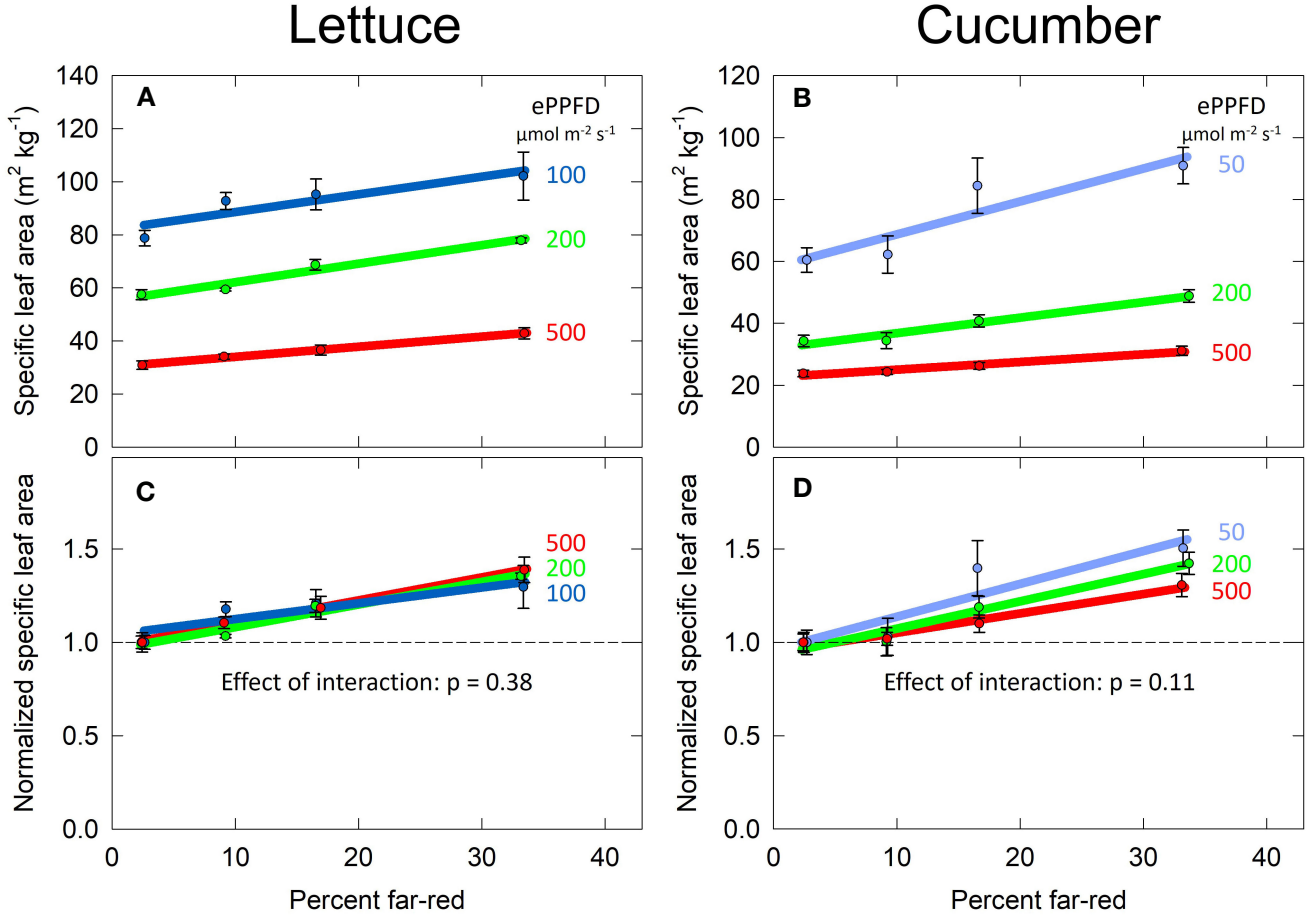
Effect of percent far-red (FR) and extended photosynthetic photon flux density (ePPFD) on leaf area in lettuce **(A, C)** and cucumber **(B, D)**. **(A, B)** represent the original values of leaf area and **(C, D)** are the normalized response, where data from each replicate in time has been normalized to its respective 2% FR control treatment for each level of ePPFD. In **(C, D)**, * indicates that the treatment is statistically different from 1 (using a student’s t-test), which represents the effect of the 2% FR control. Trend lines are included to guide the eye, and not meant to be used as a statistical representation. Error bars represent standard error for n = 3 replicates in lettuce and n = 5 replicates in cucumber.

Increases in SLA in response to both increasing percent FR and decreasing ePPFD has often been observed in previous studies. However, because there was no interaction between the two treatments to predict SLA, this parameter (SLA) does not explain the interaction between the two treatments to predict total leaf area in lettuce (**Figures 7A, C****)**.

### 3.4 Biomass partitioning

Increased shade is often reported to increase stem lengths, and this is often associated with an increase in biomass partitioning to stems. Percent stem mass, a metric that assesses partitioning to stems, is calculated by dividing stem dry mass by total shoot dry mass and multiplying by 100. Subtracting percent stem mass from 100 is the shoot biomass allocation to leaves (and cotyledons for cucumber).

**Figure 8:**
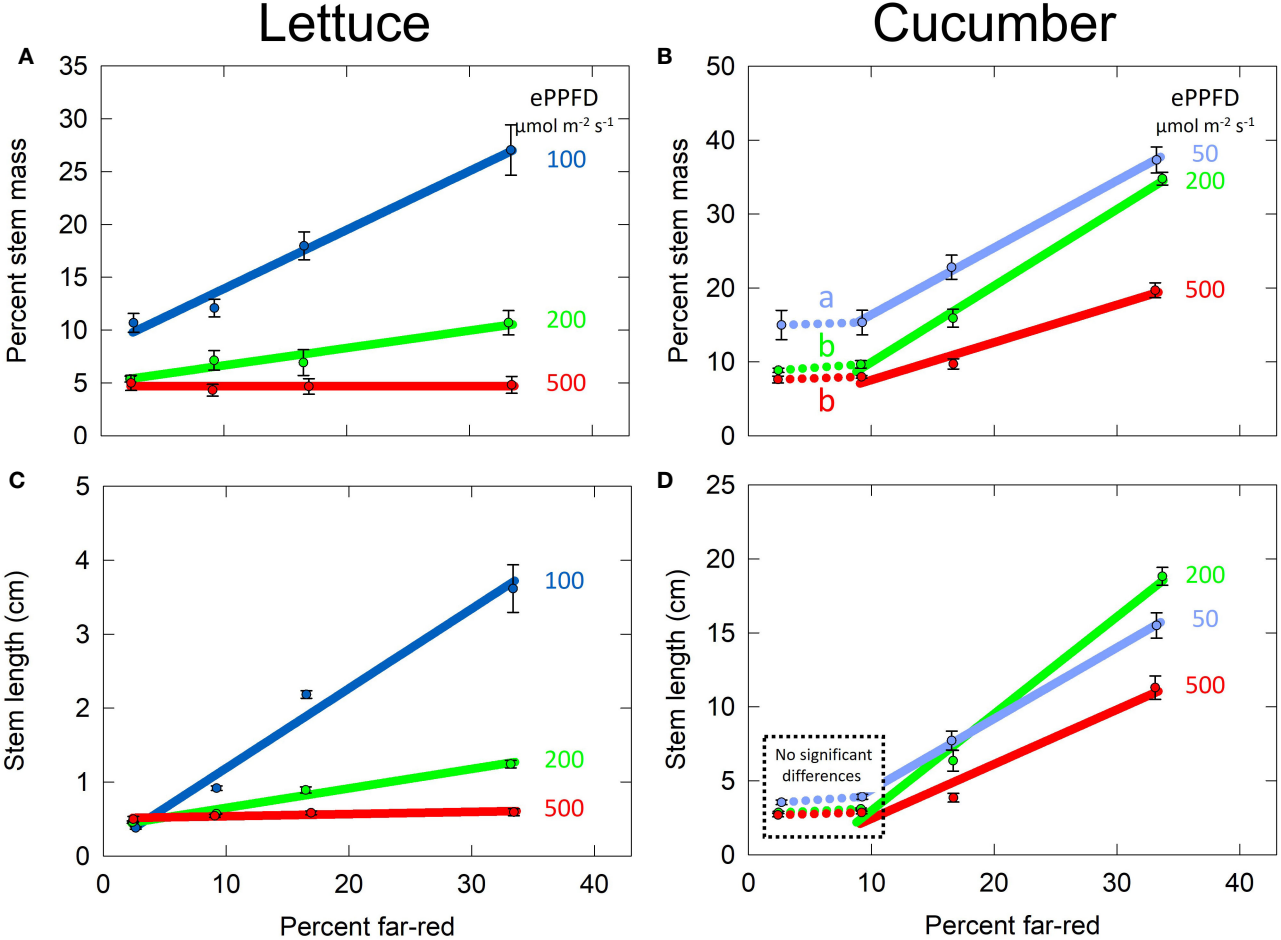
Effect of percent far-red (FR) at different levels of extended photosynthetic photon flux density (ePPFD) on stems. **(A)** Percent stem mass in lettuce, representative of biomass partitioning to stems, **(B)** percent stem mass in cucumber, **(C)** stem length in lettuce, and **(D)** stem length in cucumber. A two-way ANOVA analysis and Tukey’s *post hoc* test indicated no difference in percent stem mass between 2 and 9% FR for all three levels of ePPFD, but it was significantly higher at the lowest ePPFD for both the 2% and 9% FR treatments. The letters indicate this effect showing percent stem mass was significantly higher at 2 and 9% FR at an ePPFD of 50 μmol m^-2^ s^-1^ than 2 and 9% FR at an ePPFD of either 200 or 500 µmol m^-2^ s^-1^. Because the 2% data was removed to simplify the model and all remaining data was analysed with linear mixed-effects regression analysis. Error bars represent standard error for n = 3 replicates in lettuce and n = 5 replicates in cucumber. No significant differences

In lettuce, the effects of percent FR and ePPFD on percent stem mass were analyzed with linear mixed-effects regression following the same procedure as SLA (**Figure 6A**). In cucumber, the effect of percent FR on percent stem mass was non-linear. Using two-way ANOVA analysis and Tukey’s *post hoc* test, it was determined that there was no difference between 2 and 9% FR for the three levels of ePPFD (**Figure 8B**, dotted lines), but percent stem mass was significantly higher at the lowest ePPFD for both the 2% and 9% FR treatments (**Figure 8B**). Since there was no difference between 2 and 9% FR, to simplify the model, the 2% FR treatments were removed for analysis and the remaining data (9, 17 and 33% FR) were analyzed with linear mixed-effects regression analysis, similar to lettuce.

Increasing FR from 9 to 33% increased biomass partitioning to stems at all three levels of ePPFD in cucumber. Additionally, there was an effect of ePPFD on biomass partitioning to the stems with an increase in percent stem mass at lowest ePPFD. Finally, there was an interaction between the two treatments, meaning that there was a difference in the slopes of the lines among the levels of ePPFDs (p < 0.001). Unlike SLA, this interaction was determined for non-normalized data, although normalized data also showed an interaction (this is the case for both percent stem mass and stem length in both species). The interaction between percent FR and ePPFD resulted in a decreased response of percent stem mass to percent FR at the at the highest ePPFD (**Figure 8B**).

In lettuce, there was also an effect of percent FR, ePPFD and an interaction between the two treatments in the prediction of percent stem mass (p < 0.001). The most striking impact of the interaction between percent FR and ePPFD in lettuce was that increasing percent FR from 2 to 33% increased percent stem mass by 2.5-fold at the lowest ePPFD but had no effect at the highest ePPFD (**Figure 8A**). Similar to the effect of ePPFD at the lowest percent FR in cucumber (dotted lines in **Figure 8B**), percent stem mass in lettuce tended to be higher at 100 µmol m^-2^ s^-1^ compared to the higher two intensities at 2% FR. But, this was not statistically significant at the p = 0.05 level using Tukey’s *post hoc* test from a two-way ANOVA (p = 0.09 and 0.06 for 100 compared to 200 and 100 compared to 500 µmol m^-2^ s^-1^, respectively).

The striking interaction between percent FR and ePPFD in the prediction of percent stem mass in lettuce explains the interaction between the two treatments in the prediction of total plant leaf area. As percent stem mass (shoot biomass allocation to stems) increases, percent leaf mass (shoot biomass allocation to leaves) decreases. Because SLA increased with increasing percent FR at all levels of ePPFD (**Figure 6C**), and because biomass partitioning to leaves was unaffected by percent FR at the highest ePPFD, overall total leaf area was increased with increasing percent FR. However, at the lowest ePPFD, even though increasing FR increased SLA by about 30%, biomass accumulation was redirected away from the leaves to a significant enough degree to reduce total leaf area.

Due to thermal reversion of phytochrome, ePPFD and FR have been combined into one unifying model called the cellular model, which accounts for both increased thermal reversion at higher temperatures and the larger contribution of thermal reversion on phytochrome dynamics at lower ePPFD. This model was unable to explain the response of percent stem mass in cucumber (**Figure S3**), indicating a contribution of other factors (e.g. cryptochrome and/or photosynthate signals, see discussion).

### 3.5 Stem length

The responses of lettuce and cucumber stem lengths to percent FR and ePPFD were similar to the response of percent stem mass, and thus the data for each species was analyzed using the same methods (**Figure 8**). There was an effect of percent FR, ePPFD and an interaction between the two (p < 0.001), in the prediction of stem length in both species. In cucumber, the tallest plants at the highest percent FR occurred at the middle ePPFD instead of the lowest ePPFD. A similar response was observed in longest petiole length (**Figure S4**). This is likely a direct result of lower photosynthesis resulting in reduced growth rate.

### 3.6 Responses of other lettuce cultivars

We investigated the same five parameters (biomass accumulation, total leaf area, specific leaf area, percent stem mass, and stem length) in two additional cultivars of lettuce: ‘Waldmann’s Dark Green’ and ‘Green Salad Bowl’ (**Figures S5**–**S8**). Because ‘Green salad bowl’ was replicated the same number of times as ‘Rex’ (n = 3), it was analyzed following the same statistical approach. ‘Waldmann’s dark green’ was only replicated once, and thus, no statistical analysis was performed, but the trends are consistent.

In all three cultivars, increasing percent FR resulted in an increase in biomass accumulation at the high ePPFD, but a decrease in accumulation at the low ePPFD. At the intermediate ePPFD, ‘Waldmann’s dark green’ showed a general downward trend with increasing percent FR, while ‘Green salad bowl’ showed an increase followed by a decrease (**Figure S5**). As expected, the response of total leaf area was similar to the response of total shoot dry mass (**Figures S1**, **S6**). In general, the responses of SLA were similar between all three cultivars (**Figure S7**), although FR appeared to have a larger effect at the high ePPFD in ‘Waldmann’s dark green’, but this was likely due to reduced growth in the no added FR treatment. Additionally, at the lowest ePPFD in ‘Green salad bowl’ increasing FR appeared to increase SLA up to a threshold, then decrease it. Finally, there was a clear interaction between percent FR and ePPFD in predicting percent stem mass and stem length in all three cultivars (**Figure S8**). One notable difference from ‘Rex’ is that increasing percent FR had a small effect on percent stem mass and stem length at the high ePPFD in ‘Green salad bowl’. These results indicate that the general interaction between ePPFD and percent FR on the prediction of leaf area and biomass accumulation may be observed across many lettuce cultivars.

## 4 Discussion

Leaves are the primary organs responsible for carbon gain as these organs have higher photosynthetic capacity than stems, petioles, and fruits (Xu et al., 1997). Despite the importance of leaves, the response of total leaf area to shade signals has been inconsistent (Casal and Smith, 1989; Casal, 2012; Demotes-Mainard et al., 2016). Here, we show that the effect of percent FR on leaf area/expansion can depend on the ePPFD (**Figures 7A, C**; **S6**). We conclude that this interaction is a byproduct of the differences in biomass allocation between stems and leaves under these conditions, and not a response of SLA, which increased with FR under all levels of ePPFD (**Figures 6**; **S7**). This potential dependency of leaf expansion on biomass allocation was previously noted by Casal et al. (1987) who observed that, in contrast to many other reports at the time, *Petunia axilaris*, *Taraxacum officinale*, *Terminalia ivorensis*, and numerous grass species all showed increased leaf area at low R:FR ratios (higher fraction of FR), while stem length in these species was relatively unaffected. Although we agree that FR effects on leaf area are highly dependent on biomass partitioning between stems and leaves, we observed an increase in both leaf area and stem length with increasing percent FR in cucumber.

The simultaneous increase in leaf area and stem length in cucumber may be explained by 1) the effect of increasing FR on percent stem mass was reduced at the highest ePPFD (**Figure 8B**); and 2) there was an insignificant (p = 0.11, **Figure 6D**) trend of a larger effect of increasing percent FR on SLA at lower levels of ePPFD in cucumber. Collectively, these factors may have allowed the overall leaf area in cucumber to increase despite a simultaneous increase in stem length. Additional studies are warranted.

Increased percent stem mass was associated with increased stem length (**Figure 8**). Biomass allocation between leaves and stems has been reported to be sensitive to both FR and ePPFD (Morgan and Smith, 1979; Morgan and Smith, 1981; Ballaré et al., 1987; Poorter et al., 2012; Yang et al., 2018). Faster rates of stem elongation may require an increase in sugars to support the upregulated metabolism. As such, increases in percent FR have been associated with 1) a decrease in hypocotyl (but not cotyledon) expression of genes related to photosynthesis, the Calvin cycle, and sucrose/starch biosynthesis in *Arabidopsis*, suggesting a decrease in local sugar production in the hypocotyl and thus an increase in stem sink strength; and 2) an increase in the transport of radiolabeled carbon from cotyledons to hypocotyls (de Wit et al., 2018). This transported carbon was allocated to multiple pools of carbon within the hypocotyl including amino acids, lipids, neutral sugars, proteins and cell walls. Of these, the insoluble portion—cell walls and proteins—and the ethanol soluble portion—lipids/cell membranes—both increased over 3-fold from the high R:FR (low percent FR) to the low R:FR (high percent FR) treated seedlings. The increases in these two pools indicate an increase to structural components, meaning an increase in hypocotyl/stem biomass accumulation. In addition to this change in carbon transport, shade- induced upregulation of auxin inhibits cytokinin expression in leaf primordia (Carabelli et al., 2007), which leads to an inhibition of cell proliferation in leaves causing reduced leaf area (Riefler et al., 2006; Wu et al., 2017). This provides a second mechanism by which biomass allocation to leaves decreases in shade. In a meta-analysis, Poorter et al. (2012) concluded that low ePPFD decreases the root mass fraction while increasing the stem and leaf mass fractions, and low R:FR (high percent FR) increases the stem mass fraction with little effect on root mass fraction. We did not measure root mass fraction, and it is possible that it increased under low ePPFD, but this would not change the conclusion that increasing the percent FR had a larger effect on stem biomass partitioning at lower ePPFD.

Shade symptoms are often reported to increase in response to both decreases in photon intensity and increases in FR. The response to photon intensity is often associated with a decrease in blue photons specifically (de Wit et al., 2016; Pedmale et al., 2016), although this is not always the case (Millenaar et al., 2009). Previous studies have shown interactions between low blue (low photon intensity) and increased FR, with synergistic shade-induced stem elongation under both conditions compared to one shade condition alone (de Wit et al., 2016). These previous results are very similar to the results obtained in this study (**Figure 8**). This interaction is hypothesized to be related to both the PHYTOCHROME INTERACTING FACTOR (PIF) family of transcription factors and the E3 ubiquitin ligase formed by CONSTITUTIVELY PHOTOMORPHOGEIC 1 (COP1) and SUPRESSOR OF PHYA-105(SPA) proteins (Su et al., 2017), both of which are regulated by the phytochrome and cryptochrome photoreceptors (Legris et al., 2019).

The highest FR treatments in the two highest ePPFD treatments had overlapping leaves resulting in inter-plant competition for light and thus decreased growth rates of individual plants. This leaf overlap means that the effect of FR would be underestimated if the results were applied to single spaced plants. This may explain the saturated effect of FR on leaf expansion in lettuce between 9 and 33% FR at the highest ePPFD treatment (**Figure 7**). However, the increased shade signals induced by the plant competition would have continued to increase SLA (**Figure 6**). A similar response was observed in the lowest ePPFD treatments: the effect of FR on leaf expansion saturated between 9 to 33% FR, but SLA, stem length and percent stem mass continued to increase in response to FR (**Figures 7****,** **8**). In this case, plant competition does not explain the saturation, and therefore there may be some minimum leaf expansion that the plant maintains to allow for some photon capture and continued elongation.

### 4.1 Ecological significance

Ballaré et al. (1987) demonstrated that in competitive light environments FR can increase in the horizontal direction prior to a decrease in PAR. This is notable because it means that in certain circumstances there can be increases in FR in the natural environment even at high PAR. These FR signals are the first indication of oncoming shade and they have been shown to increase internode elongation prior to the subsequent decrease in PAR in *Sinapis alba* and *Datura ferox* (Ballaré et al., 1987). These two species have an upright plant architecture, similar to cucumber, in comparison to the rosette architecture of lettuce and *Arabidopsis*. Perhaps plants with an upright architecture, like cucumber, *Sinapis alba*, and *Datura ferox*, will always elongate in response to increasing FR no matter the ePPFD. We did not investigate the highest intensities that occur in the middle of a summer day, above 2000 µmol m^-2^ s^-1^, but perhaps there would have been no effect of FR on stem elongation in cucumber at this intensity. It is interesting that cucumber increased leaf area at all three levels of ePPFD, meaning that in the natural environment FR would be likely to increase leaf expansion from both neighboring plants at high ePPFD and from true shade at low ePPFD.

For lettuce, and perhaps other species with a rosette architecture, the increase in FR from neighboring vegetation could actually increase leaf expansion in ecosystems with more light. This is further notable because in this study we substituted PAR for FR, but in the natural environment FR can increase with no change in PAR (Ballaré et al., 1987), and this could lead to additional increases in growth through photosynthesis. However, the increase in FR in most shaded environments would be much more likely induce decreases in leaf expansion, as the plants attempt to reach out of the shade.

The lowest two FR treatments (2 and 9%) are probably not entirely comparable to sunlight. Leaf expansion was not significantly different between 17 and 33% FR; thus, it could be argued that no such increases in leaf expansion would occur in the natural environment. However, FR LEDs have a peak that closely matches the peak of the Pfr form of phytochrome. Therefore, lower percentages of FR from FR LEDs may be comparable to higher percentages of FR in sunlight, but such comparisons are difficult.

### 4.2 Photosynthetic considerations

Often, studies investigating plant responses to FR typically add FR photons on top of a constant intensity of PAR instead of decreasing the photon intensity within PAR while increasing FR. Maintaining a constant ePPFD removes the potential impact of photosynthesis on leaf expansion. Assuming PAR and FR photons both drive photosynthesis with equal efficiencies, then the studies performed here (substituting PAR with FR) would at most result in equal leaf-level photosynthesis. However, this 1:1 assumption is likely not the case. Zhen and Bugbee (2020a) noted that the photosynthetic relationship of PAR photons and FR photons began to deviate from a 1:1 equivalency when FR was added in excess of 40% of PAR (29% FR based on the definition used here). Furthermore, Zhen et al. (2019) showed that photons at 752 nm did not increase the quantum yield of PSII (the fraction of absorbed photons used for photochemistry), indicating that photons at 752 nm did not contribute to photosynthesis (the next lowest wavelength measured was 731 nm). These considerations indicate that 1) extending the definition of photosynthetic photons out to 750 nm may overestimate the photosynthetic response, and b) 33% FR used in this study would have deviated from a 1:1 photosynthetic relationship FR and PAR. Perhaps this also explains the saturating effect of FR on shoot dry mass and leaf area (**Figures 5**, **7**).

Despite this limitation of our experiment, both of these factors indicate that the higher FR treatments in this study would have resulted in lower leaf level photosynthesis, which further highlights the effect increasing percent FR can have on leaf expansion and thus photon capture in cucumber and lettuce (at high photon fluxes in the latter).

### 4.3 Implications for horticulture

The results presented here have substantial implications for the horticultural industry – especially in sole-source lighting environments, where electric lighting from LEDs has been rapidly expanding. Providing photons for crop production is expensive, especially when considering the electrical power required to operate the LEDs. FR LEDs are among the highest efficacy LEDs (µmol of photons per joule input electrical energy), primarily because of the lower energy of the photons (Kusuma et al., 2020). Interest in FR for horticultural environments has expanded in the past decade where studies have often found that adding FR increases leaf area or plant diameter in lettuce, resulting in an increase in fresh and dry mass (Lee et al., 2016; Meng and Runkle, 2019; Zou et al., 2019; Zhen and Bugbee, 2020b; Legendre and van Iersel, 2021). But, many of these studies supplemented FR rather than substituting PAR photons for FR. The addition of FR LEDs in fixtures increases costs for two reasons: 1) FR LEDs are in lower demand, meaning they are more expensive, and 2) increasing the total photon output of a fixture increases the power requirement. Thus, substitution of PAR with FR is much more reasonable for practical applications. Zhen and Bugbee (2020b) found that substituting 14% of PAR with FR at an ePPFD of 350 µmol m^-2^ s^-1^ resulted in similar quantum yields for CO_2_ fixation in ‘Waldmann’s dark green’ lettuce, but increased biomass accumulation by about 30% *via* an increase in photon capture (leaf expansion). We observed a similar response with ‘Waldmann’s dark green’ in our experimental chambers; adding 9, 17 and 33% FR at an ePPFD of 500 µmol m^-2^ s^-1^ resulted in 31 to 33% increase in biomass accumulation. However, at lower ePPFD (100 and 200 µmol m^-2^ s^-1^), increasing percent FR resulted in a decrease in biomass accumulation (**Figures S5**-**S8**).

In another similar study, Legendre and van Iersel (2021) concluded that FR photons were between 57 to 183% more effective at increasing photon capture than PAR photons in ‘Green salad bowl’ lettuce and were thus 93 to 162% more effective at increasing biomass accumulation. Interestingly, they observed no interaction between PPFD and FR when PPFD varied between 111 and 245 µmol m^-2^ s^-1^. In this cultivar, we observed an increase in leaf expansion and shoot dry mass with an increase in percent FR at 500 µmol m^-2^ s^-1^, but a decrease at an ePPFD of 100 µmol m^-2^ s^-1^ and little effect at 200 µmol m^-2^ s^-1^ (**Figures S5**, **S6**). Interestingly at 200 µmol m^-2^ s^-1^ shoot dry mass was higher at 9% FR compared to 2% FR (**Figure S5F**), but at higher levels of FR, dry mass decreased. The contradictions between our study and Legendre and van Iersel (2021) are difficult to parse, as many of the environmental conditions were similar.

Producers can increase yields of lettuce by including FR LEDs in lighting fixtures because FR photons will increase leaf expansion and photon capture, while decreasing electrical input (depending on operating conditions and other LEDs in the fixture). However, caution must be taken as increases will only occur at high enough ePPFD. For cucumber, including more FR appears to be beneficial no matter the ePPFD, but the increase in leaf expansion appears to be accompanied by increases in stem elongation. This may be manageable in greenhouse environments but may be undesirable in sole-source lighting environments. Additionally, the added FR may only benefit cucumber plants when they are young.

Based on current definitions, FR photons are not considered in the calculation of photosynthetic photon efficacy, which is the flux of photosynthetic photons (µmol s^-1^) divided by the input power (W), resulting in µmol per J. Currently, photosynthetic photons are considered to only be those with wavelengths between 400 to 700 nm based on studies by McCree (1972a); McCree (1972b). Because this definition excludes photons between 700 to 750 nm, the benefits of photons from FR LEDs, both on leaf expansion and photosynthesis are excluded. These definitions ought to be reconsidered (Zhen et al., 2021), but at the same time, FR must be used with caution, as we have shown it can be detrimental in some conditions.

## Data availability statement

The raw data supporting the conclusions of this article will be made available by the authors, without undue reservation.

## Author contributions

PK and BB contributed to the design of the study, analysis of data, and writing of the manuscript. Both authors approved the submitted version.
