# Supplementary material for: On the contrasting morphological response to far-red at high and low photon fluxes
Source: Front Plant Sci. 2023 Jun 2;14:1185622. doi: 10.3389/fpls.2023.1185622 (PMC10274578; doi:10.3389/fpls.2023.1185622)
Supplement: Supplementary file 1 [file DataSheet_1.docx]

SUPPLEMENTAL MATERIAL**
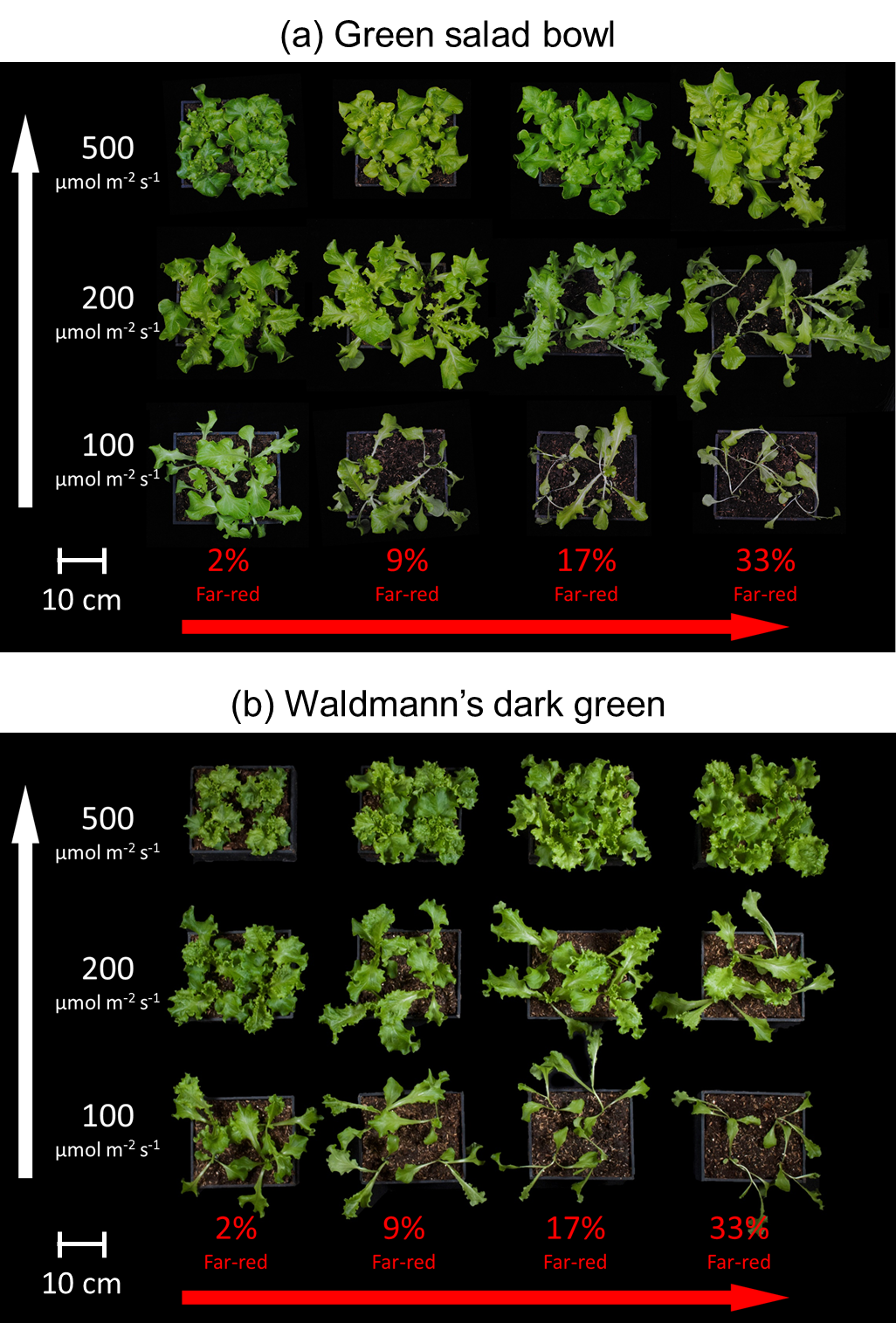
**

**Fig. S1:** Overhead view of lettuce plants from all the treatments in one replicate for (a) ‘Waldmann’s dark green’ and (b) ‘Green salad bowl’. The white arrow on the left indicates increasing extended photosynthetic photon flux density (ePPFD) and the red arrow on the bottom indicates increasing percent far-red.


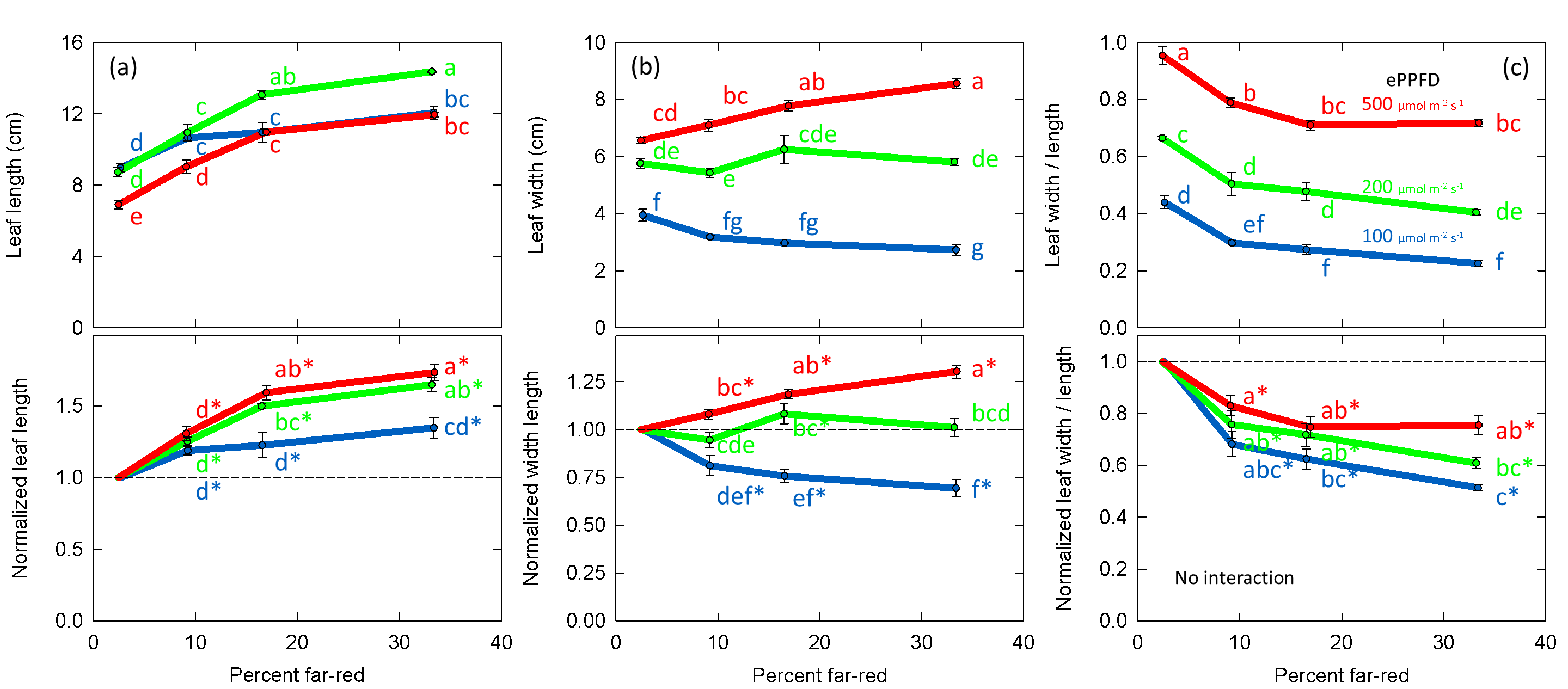


**Fig. S2:** Effect of percent far-red (FR) and extended photosynthetic photon flux density (ePPFD) on leaf shape in lettuce. (a) Leaf length (b) leaf width and (c) leaf width divided by leaf length. Top graphs are the original values while bottom graphs are the normalized response, where data from each replicate in time has been normalized to its respective 2% FR control treatment for each level of ePPFD. In the normalized graphs, * indicates that the treatment is statistically different from 1 (using a student’s t-test), which represents the effect of the 2% FR control. Error bars represent standard error for n = 3 replicates. A higher leaf width/length ratio indicates a rounder leaf while a lower ratio indicates a longer, narrower leaf.


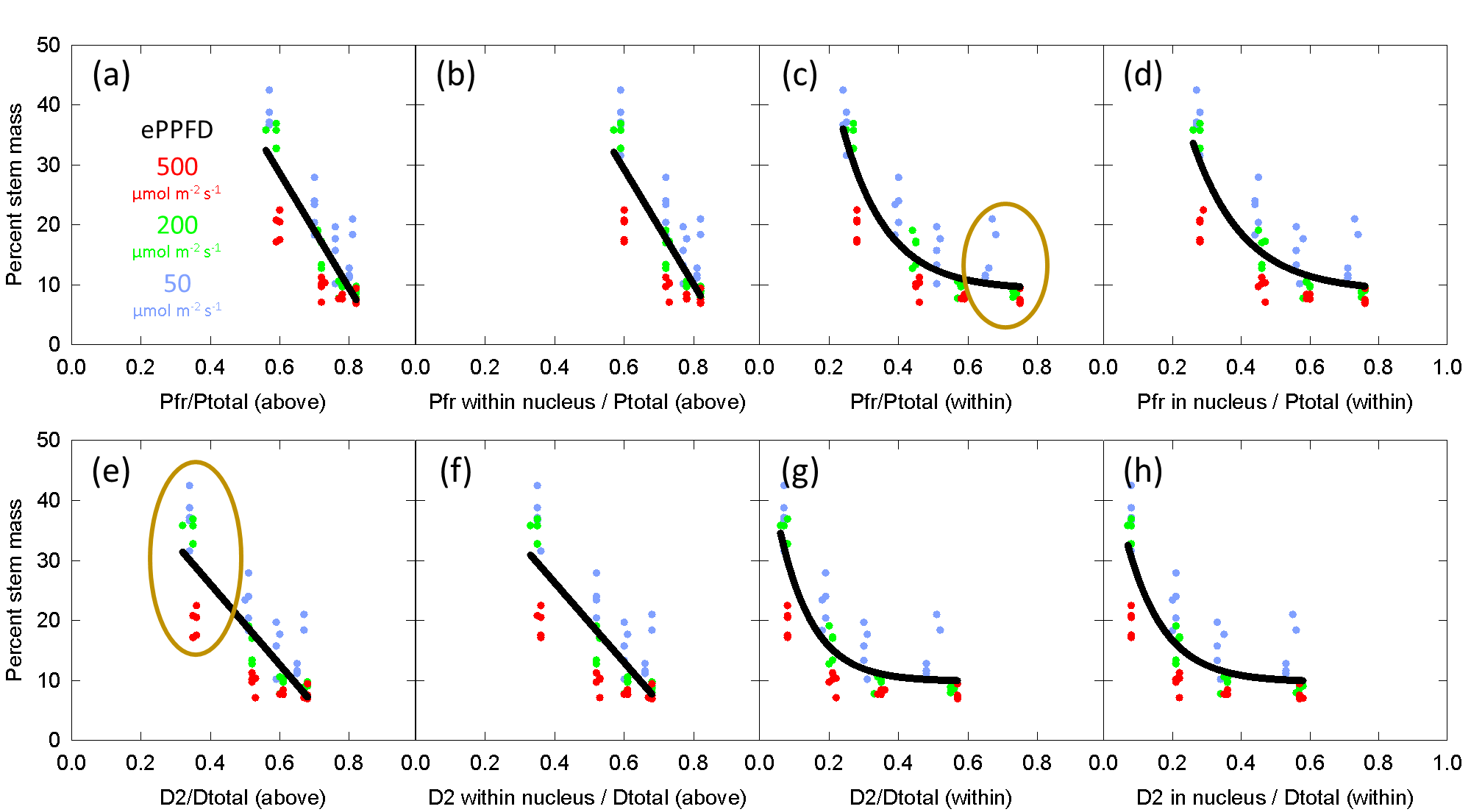


**Fig. S3:** Model of the effect of percent far-red (FR) and extended photosynthetic photon flux density (ePPFD) on percent stem mass in cucumber using the *cellular model* developed by Rausenberger et al. (2010), Klose et al. (2015), Sellaro et al. (2019), and Smith and Fleck (2019). For each graph, the program provided by Smith and Fleck (2019) was used to calculate the size of different pools of phytochrome as a fraction of the total pool of phytochrome, given the average spectral photon distribution (SPD) of each replicate. Then, the average percent stem mass of the four plants in each treatment were plotted. Linear or exponential decay models were used to fit all the data.

In (a) through (d) Pfr is assumed to be the active form of phytochrome, while in (e) through (h), D2 (the fully active Pfr-Pfr homodimer) is assumed to be the active form. In (a), (b), (e) and (f) the SPD above the leaf is input into the model, while in (c), (d), (g) and (h), spectral distortion functions are first used to estimate the SPD within the leaf.

In (c), the dark yellow circle shows the relative decrease in Pfr at lower ePPFD compared to higher ePPFD. This is caused by a greater significance of thermal reversion at lower photon intensities. In (d) the dark yellow circle shows the spread of the data between different levels of ePPFD. This shows that thermal reversion did not shift the expected response at lower intensities enough make the data fall on a single line. This strongly indicates that some other factor (e.g. blue photons) were contributing to the response.


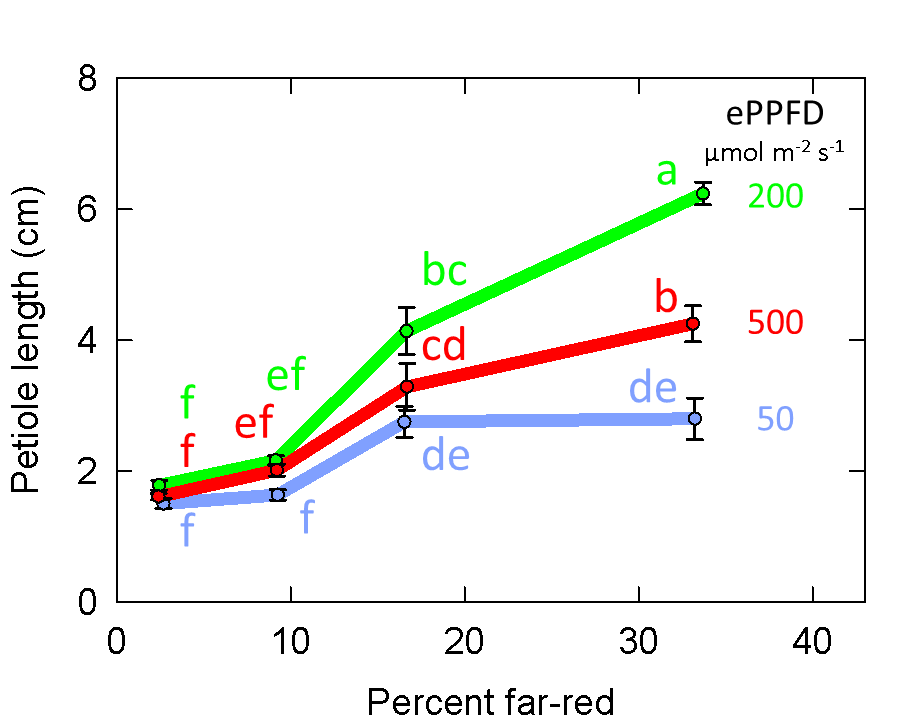


**Fig. S4:** Effect of percent far-red (FR) and extended photosynthetic photon flux density (ePPFD) on petiole length in cucumber. Error bars represent standard error for n = 5 replicates in cucumber.


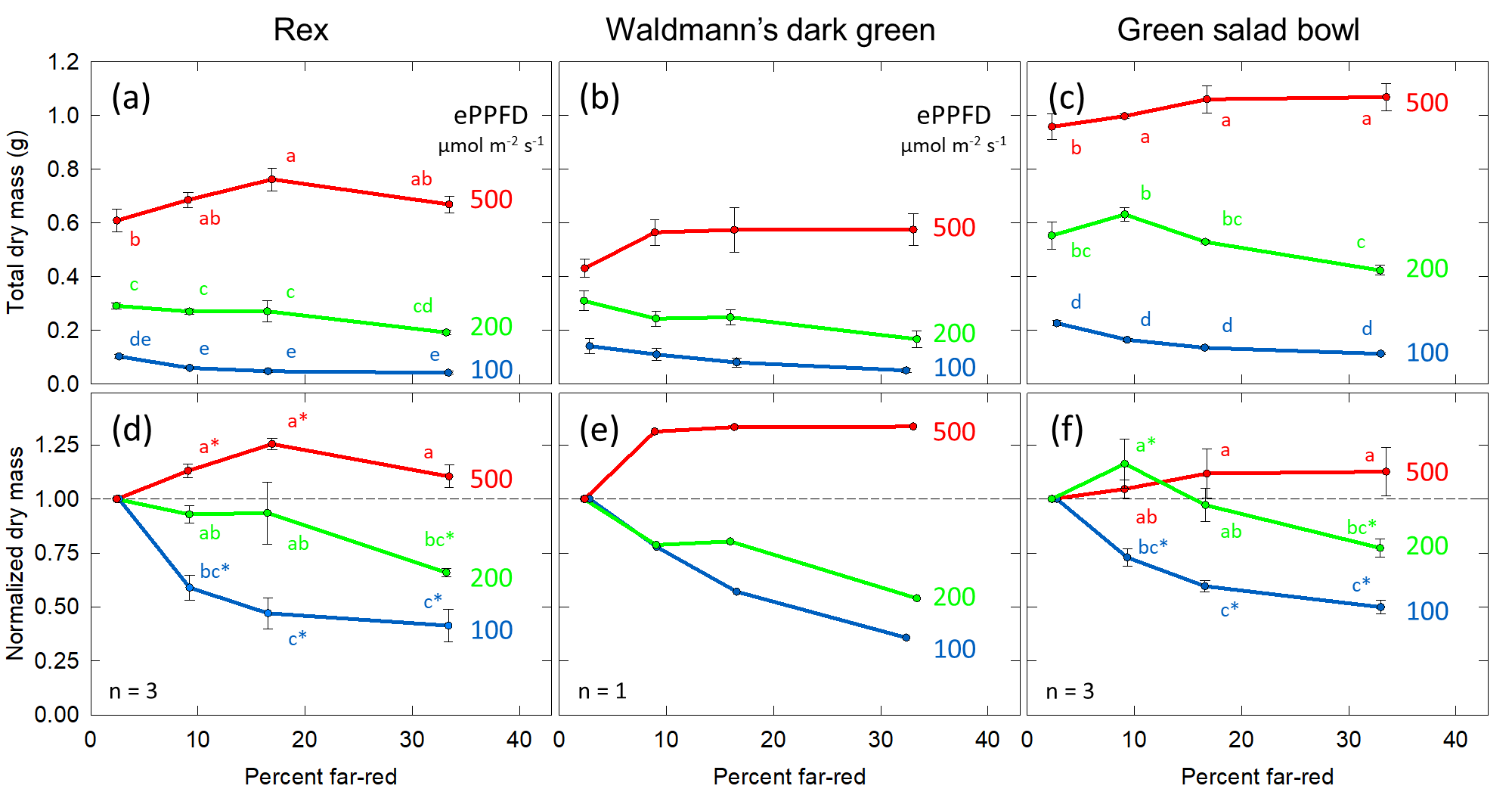


**Fig. S5:** Effects of percent far-red (FR) and extended photosynthetic photon flux density (ePPFD) on shoot dry mass in ‘Rex’ (a,d), ‘Waldmann’s dark green’ (b,e) and ‘Green salad bowl’ (c,f). (a – c) represent the original values of dry mass and (d – f) are the normalized response, where data from each replicate in time was normalized to its respective 2% FR (no added FR) control treatment for each ePPFD. In (d,f), * indicates that the treatment is statistically different from 1 (using a student’s t-test), which represents the response of the 2% FR control. In (a,c,d,f), error bars represent standard error for n = 3 replicates. In (b) error bars represent the standard error of individual four plants in the single replicate study. In (e), no error bars are provided due to the method of calculation. Because only one replicate study (with four plants) was performed in ‘Waldmann’s dark green’, no statics were performed on this data. Data in (a,d) are the same as the data in Fig. 5a,c. Although the 17 and 33% FR at an ePPFD of 500 µmol m^-2^ s^-1^were not determined to be statistically different from 1 in (f), the p-values for these treatments were 0.072 and 0.060, respectively.


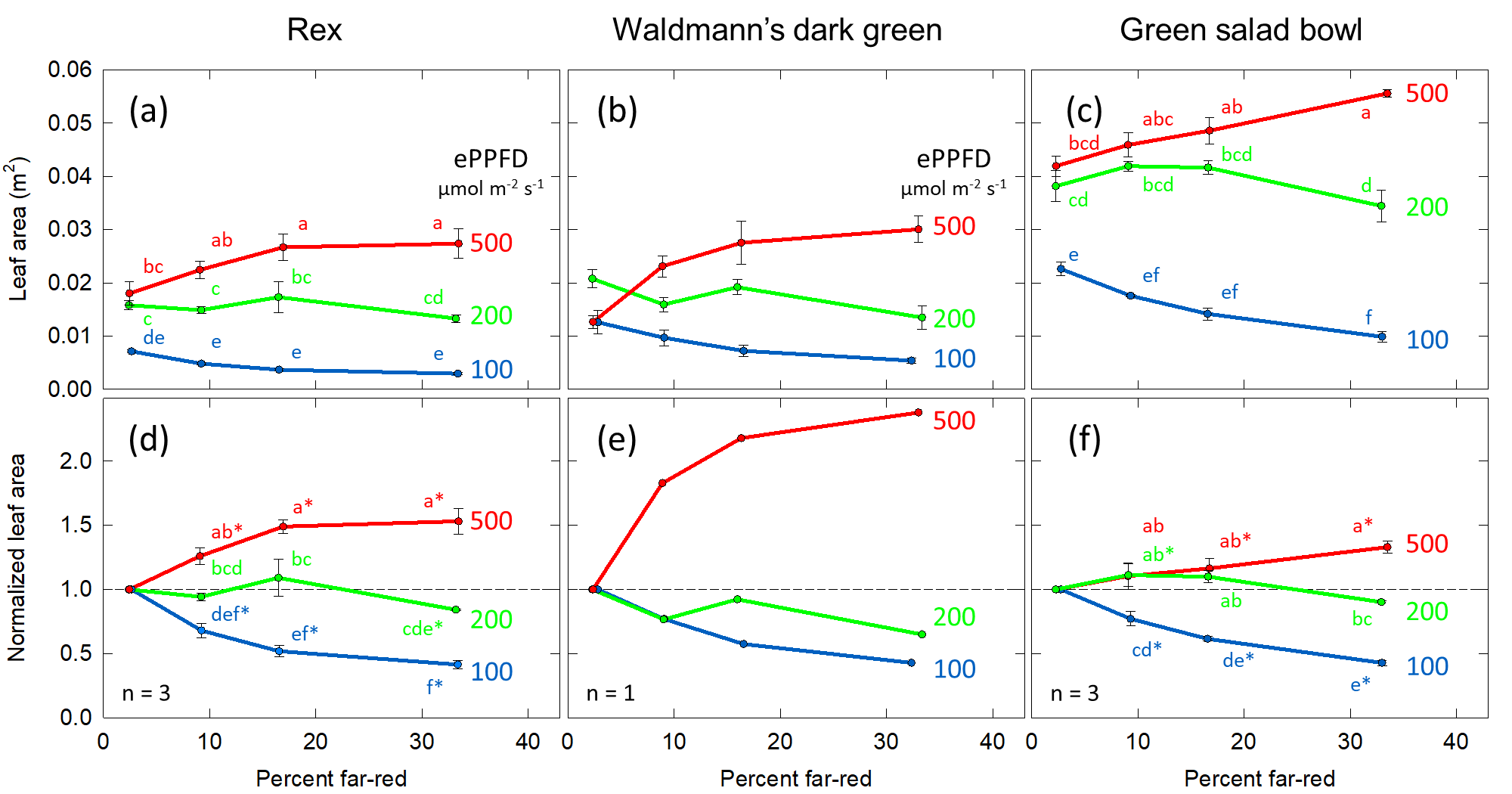


**Fig. S6:** Effects of percent far-red (FR) and extended photosynthetic photon flux density (ePPFD) on leaf area in ‘Rex’ (a,d), ‘Waldmann’s dark green’ (b,e) and ‘Green salad bowl’ (c,f). (a – c) represent the original values of dry mass and (d – f) are the normalized response, where data from each replicate in time was normalized to its respective 2% FR (no added FR) control treatment for each ePPFD. In (d,f), * indicates that the treatment is statistically different from 1 (using a student’s t-test), which represents the response of the 2% FR control. In (a,c,d,f), error bars represent standard error for n = 3 replicates. In (b) error bars represent the standard error of individual four plants in the single replicate study. In (e), no error bars are provided due to the method of calculation. Because only one replicate study (with four plants) was performed in ‘Waldmann’s dark green’, no statics were performed on this data. Data in (a,d) are the same as the data in Fig. 6a,c. Although the 9% FR at the ePPFD of 500 µmol m^-2^ s^-1^ and the 17 and 33% FR at the ePPFD of 200 µmol m^-2^ s^-1^ were not determined to be statistically different from 1 in (f), the p-values for these treatments were 0.056, 0.064 and 0.066, respectively.


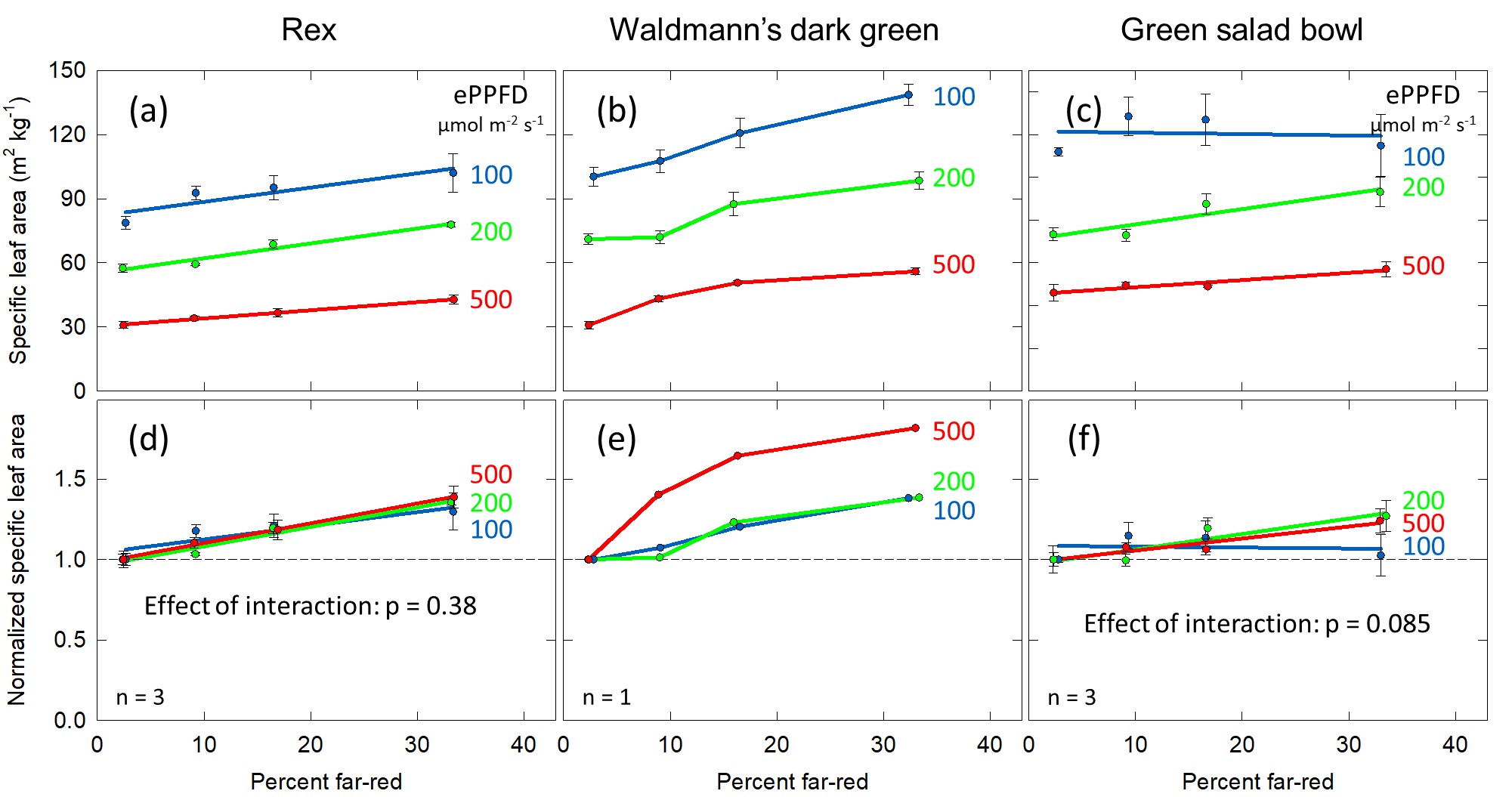


**Fig. S7:** Effects of percent far-red (FR) and extended photosynthetic photon flux density (ePPFD) on specific leaf area (SLA) in ‘Rex’ (a,d), ‘Waldmann’s dark green’ (b,e) and ‘Green salad bowl’ (c,f). (a – c) represent the original values of dry mass and (d – f) are the normalized response, where data has been normalized to the average response in the 2% FR control treatment for each ePPFD. Data in (a,d) are the same as the data in Fig. 7a,c. Decreasing the ePPFD increased SLA in both ‘Rex’ and ‘Green salad bowl’. In ‘Green salad bowl’ the effect of percent FR on SLA was not significant in the non-normalized data (p = 0.058), but it was significant in the normalized data (p = 0.005). Because only one replicate study (with four plants) was performed in ‘Waldmann’s dark green’, no statics were performed on this data. Error bars represent standard error for n = 3 replicates in (a,c,d,f), and n = 4 replicate plants in (b).


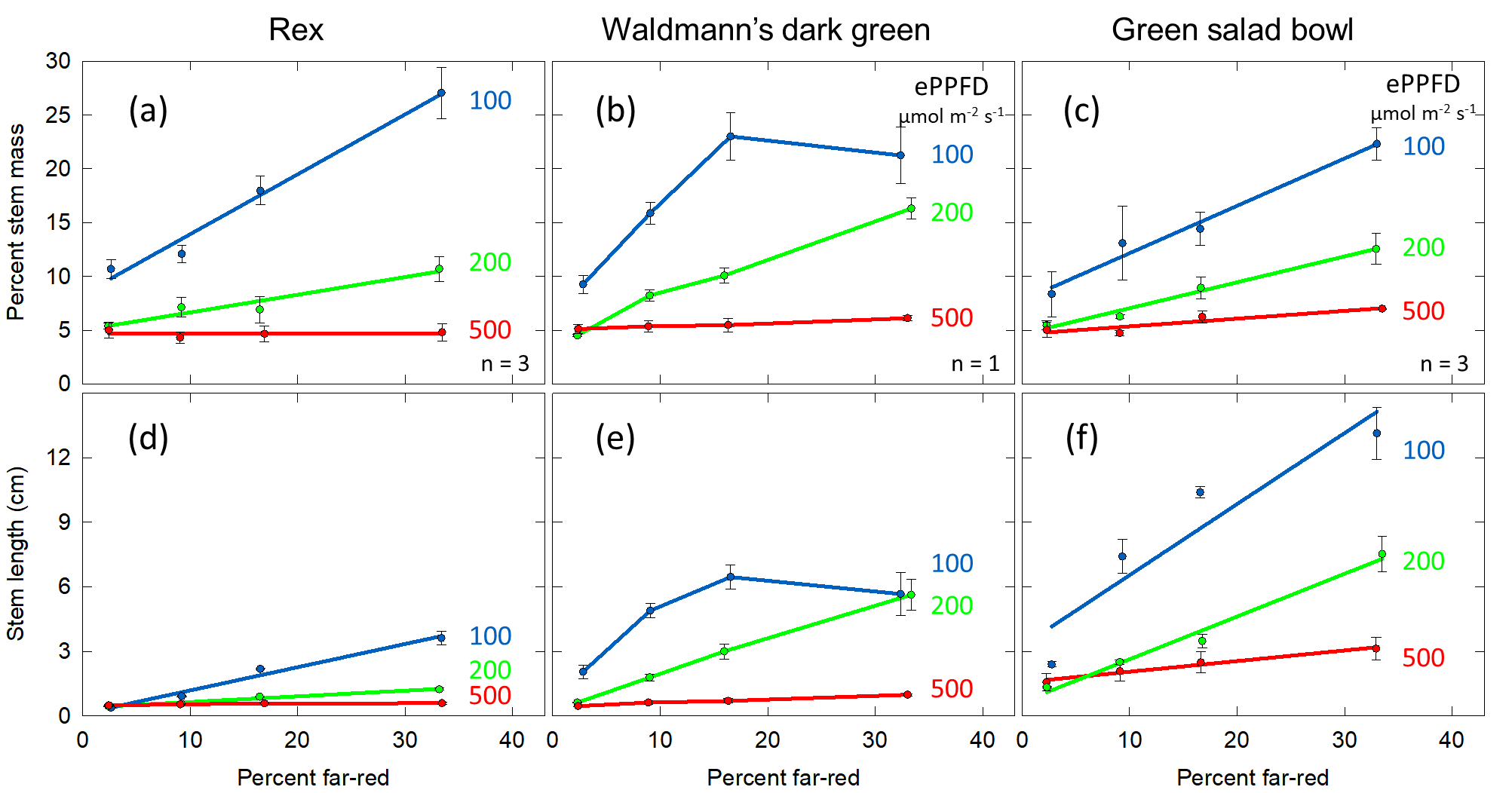


**Fig. S8:** Effect of percent far-red (FR) at different levels of extended photosynthetic photon flux density (ePPFD) on percent stem mass (a – c) and stem length (d – f) in ‘Rex’ (a,d), ‘Waldmann’s dark green’ (b,e) and ‘Green salad bowl’ (c,f). Data in (a,d) are the same as the data in Fig. 8a,c. Data in (a,c,d,f) were analyzed with linear mixed-effects regression analysis. Because only one replicate study (with four plants) was performed in ‘Waldmann’s dark green’, no statics were performed on this data. Error bars represent standard error for n = 3 replicates in (a,c,d,f), and n = 4 replicate plants in (b).
